# Harmol promotes α-synuclein degradation and improves motor impairment in Parkinson’s models via regulating autophagy-lysosome pathway

**DOI:** 10.1038/s41531-022-00361-4

**Published:** 2022-08-06

**Authors:** Jie Xu, Yun-Lin Ao, Chunhui Huang, Xiubao Song, Guiliang Zhang, Wei Cui, Yuqiang Wang, Xiao-Qi Zhang, Zaijun Zhang

**Affiliations:** 1 Guangdong Provincial Engineering Research Center for Modernization of TCM, Guangdong Provincial Key Laboratory of Pharmacodynamic Constituents of TCM and New Drug Research, College of Pharmacy, Jinan University, Guangzhou, 510632 P. R. China; 2 Institute of New Drug Research, College of Pharmacy, Jinan University, Guangzhou, 510632 P. R. China; 3 Department of Rehabilitation, the First Affiliated Hospital, Jinan University, Guangzhou, 510630 China; 4 Ningbo Key Laboratory of Behavioral Neuroscience, Zhejiang Provincial Key Laboratory of Pathophysiology, School of Medicine, Ningbo University, Ningbo, 315211 China

**Keywords:** Neurodegenerative diseases, Parkinson's disease

## Abstract

The abnormal accumulation of α-synuclein (α-syn) is a crucial factor for the onset and pathogenesis of Parkinson’s disease (PD), and the autophagy-lysosome pathway (ALP) contributes to α-syn turnover. AMP-activated protein kinase (AMPK) and the mammalian target of rapamycin (mTOR) regulate autophagy by initiating the macroautophagy cascade and promoting lysosomal biogenesis via increased transcription factor EB (TFEB) activity. Hence, activation of AMPK-mTOR-TFEB axis-mediated autophagy might promote α-syn clearance in PD. Harmol is a β-carboline alkaloid that has been extensively studied in a variety of diseases but rarely in PD models. In this study, we aimed to evaluate the effect and underlying mechanism of harmol in PD models in vitro and in vivo. We show that harmol reduces α-syn via ALP in a dose- and time-dependent manner in cell model that overexpressed human A53T mutant α-syn. We also demonstrate that harmol promotes the translocation of TFEB into the nucleus and accompanies the restoration of autophagic flux and lysosomal biogenesis. Importantly, harmol improves motor impairment and down-regulates α-syn levels in the substantia nigra and prefrontal cortex in the α-syn transgenic mice model. Further studies revealed that harmol might activate ALP through AMPK-mTOR-TFEB to promote α-syn clearance. These in vitro and in vivo improvements demonstrate that harmol activates the AMPK-mTOR-TFEB mediated ALP pathway, resulting in reduced α-syn, and suggesting the potential benefit of harmol in the treatment of PD.

## Introduction

Parkinson’s disease (PD) is the second most common neurodegenerative disease, clinically characterized by motor abnormalities (rest tremor, rigidity, bradykinesia, shuffling gait, and postural instability) and non-motor symptoms (depression, anxiety, dementia, sleep disorder)^1^. This characteristic movement restriction reduces the ability of PD patients to work, and lessens their quality of life. Indeed, late-stage PD patients often cannot take care of themselves, which significantly burdens individual caretakers, families, and society^2^. The loss of dopaminergic neurons (DAs) in the substantia nigra pars compacta (SNpc) and the presence of abnormal α-synuclein (α-syn) aggregates (Lewy bodies) are the principal pathological hallmarks of PD^3^. Misfolded and aggregated α-syn may lead to different pathogenic effects, including induction of oxidative stress^4^, increase of membrane permeability^5^, interruption of axonal transport^6^, synaptic dysfunction^7^, mitochondrial dysfunction^8^, and inhibition of the autophagy-lysosome pathway (ALP)^9^ and the ubiquitin-proteasome system (UPS)^10^. Therefore, α-syn is widely considered to play a key role in the development of PD.

ALP is a bulk degradation process that occurs in all eukaryotic cells and is mediated by lysosomes^11^. Defects of ALP result in the aggregation of α-syn in PD^12^, and pharmacological enhancement of autophagy reduces the accumulation of α-syn and neurodegenerative pathology in cellular and animal models^13^. Accumulating evidence indicates that the AMP-activated protein kinase (AMPK) promotes autophagy by sensing cellular energy status to maintain energy homeostasis^14^. An important cell-growth regulator that integrates growth factor and nutrient signals are the mammalian target of rapamycin (mTOR)^15^. Inhibition of mTOR activates ALP function and induces autophagic degradation to produce protective effects in the PD model^16^. Indeed, mTOR and AMPK act synergistically to control autophagy induction^17^. Recently, the transcription factor EB (TFEB) was identified as a master regulator of the ALP and is controlled by mTOR signaling^18^. The enhancement of TFEB stimulates ALP function and attenuates the pathology of α-syn^19^. Therefore, activation of AMPK-mTOR-TFEB axis-mediated autophagy may promote α-syn clearance and be a promising strategy for PD treatment.

Current therapies for PD include dopaminergic medications (e.g., dopamine precursors, dopamine receptor agonists, monoamine oxidase inhibitors, catecholamines, and methyltransferase inhibitors) and nondopaminergic approaches (e.g., selective serotonin reuptake inhibitors and cholinesterase inhibitors). Unfortunately, the current strategies produce serious side effects and only reduce motor and non-motor symptoms in PD patients^20^. Therefore, neuroprotective and/or disease-modifying drugs that prevent or delay PD progression are urgently needed.

**Fig. 1:**
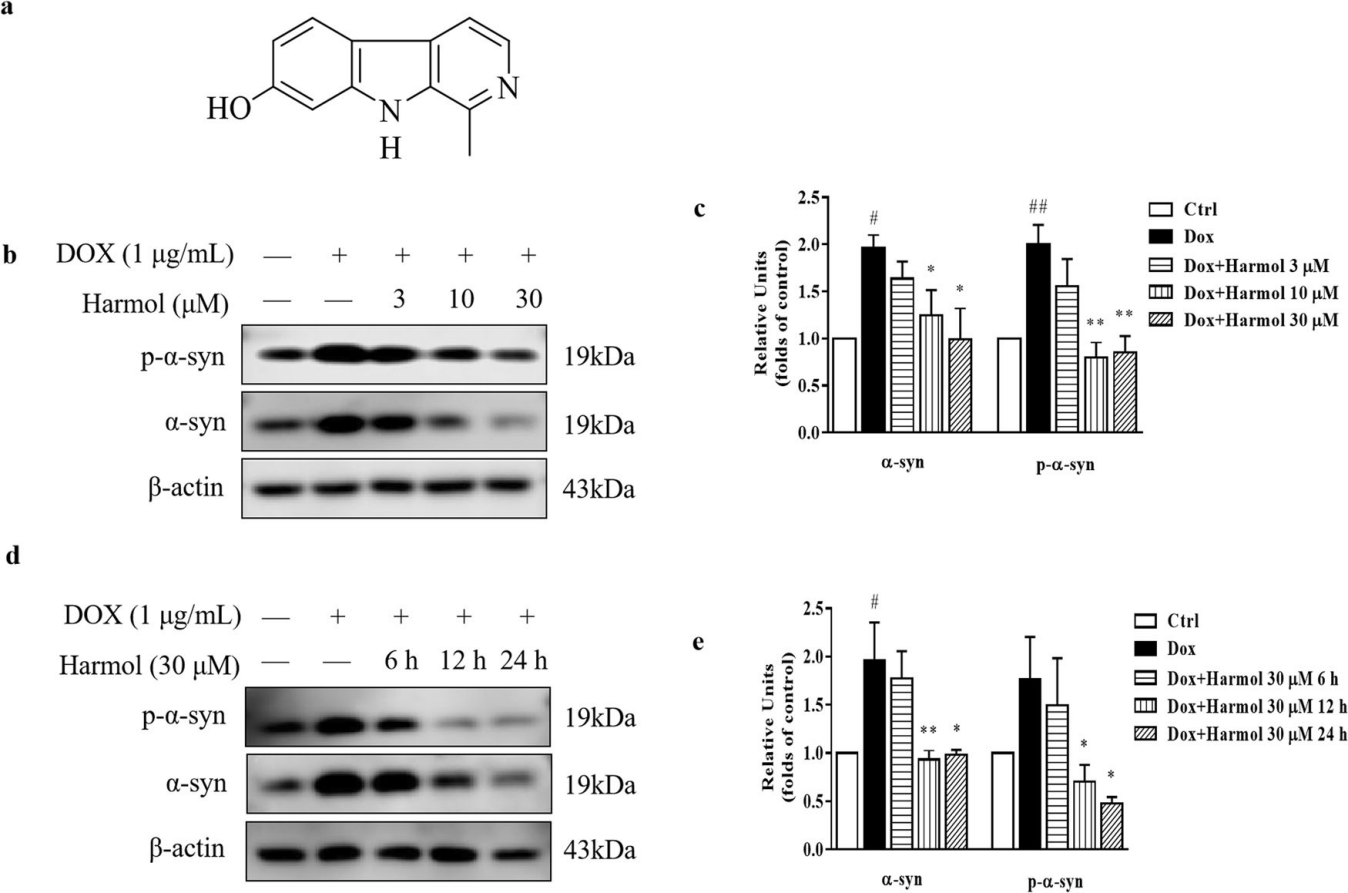
Harmol degrades the α-syn. **a** Chemical structure of harmol. **b** Tet-on inducible PC12 cells were treated with 1 µg/mL DOX for 24 h, then 3, 10, and 30 µM harmol were added for 24 h. The expression of p-α-syn and α-syn were determined by western blot. Representative blots are shown. **c** Relative intensity was normalized to that of β-actin. Data are presented as the mean ± SEM from three independent experiments. ^#^*P* < 0.05 and ^##^*P* < 0.01 vs. the control (0.1% DMSO). ^*^P < 0.05 and ^**^P < 0.01 vs. the DOX. **d** After induction with DOX for 24 h, PC12 inducible cells were incubated with harmol (30 µM) for 6 h, 12 h, and 24 h. The expression of p-α-syn and α-syn were determined by western blot. Representative blots are shown. **e** Relative intensity was normalized to that of β-actin. Data are presented as the mean ± SEM from three independent experiments. ^#^*P* < 0.05 vs. the control (0.1% DMSO). ^*^P < 0.05 and ^**^P < 0.01 vs. the DOX.

Harmol (Fig. 1a), a β-carboline alkaloid, exhibits a variety of bioactivities including antifungal^21^, antitumoral^22^, antiviral^23^, antioxidant^24^, and neuroprotective properties^25^. Harmol also inhibits human monoamine oxidase (MAO), which is linked to antidepressant effects^26^. Harmol-induced autophagy involves the protein kinase B PKB (Akt)/mTOR pathway in U251MG human glioma cells^27^. Furthermore, the expression of autophagy-related proteins and genes were regulated with increases in harmol dosage in insect Sf9 cells^28^. Therefore, we hypothesize that harmol may play a role in PD through autophagy-related pathways.

In the present study, we exhibit the novel therapeutic intervention of harmol as an autophagy enhancer. Harmol promotes the degradation of α-syn in a dose- and time-dependent manner in vitro, and ALP is involved in the mechanism of harmol for the treatment of PD. Harmol promotes the nuclear translocation of TFEB, accompanied by the restoration of autophagic flux and lysosomal biogenesis. In vivo, harmol improves motor deficits and attenuates α-syn load in the substantia nigra and prefrontal cortex. Further studies revealed that harmol promotes α-syn clearance via AMPK-mTOR-TFEB axis-mediated ALP activation. Finally, inhibition of AMPK blocks harmol-induced autophagy activation and α-syn clearance, indicating that harmol enhances autophagy via AMPK activation resulting in decreased α-syn. These findings suggest that harmol, as a new autophagy enhancer, may have the therapeutic potential for neurodegenerative diseases related to ALP dysfunction and abnormal protein accumulation.

## Results

### Harmol degrades the α-syn in vitro

The Tet-on system is an inducible gene expression system for mammalian cells. In the presence of doxycycline (DOX), the Tet-on PC12 cells overexpress α-syn under the control of a TRE3G promoter^29^. Tet-on A53T α-syn inducible PC12 cells were treated with 1 µg/mL DOX for 24 h to induce the expression of A53T α-syn. DOX was then replaced with a fresh medium to stop the α-syn expression, and different concentrations of harmol were added for another 24 h. We first determined the cytotoxicity of harmol by 3-[4,5-dimethylthiazol-2-yl]-2,5-diphenyltetrazolium bromide (MTT) analysis (Supplementary Fig. 1). Nontoxic concentrations of harmol were used for subsequent tests. Western blot results demonstrate a significant dose-dependent reduction of phosphorylated (p-) and total α-syn after harmol treatment (Fig. 1b, c). Meanwhile, harmol (30 μM) time-dependently promotes p-α-syn and α-syn clearance (Fig. 1d, e) in PC12 inducible cells and demonstrates pro-degradation activity between 6 h and 24 h.

### Harmol promotes the degradation of α-syn through ALP and the nuclear translocation of TFEB

**Fig. 2:**
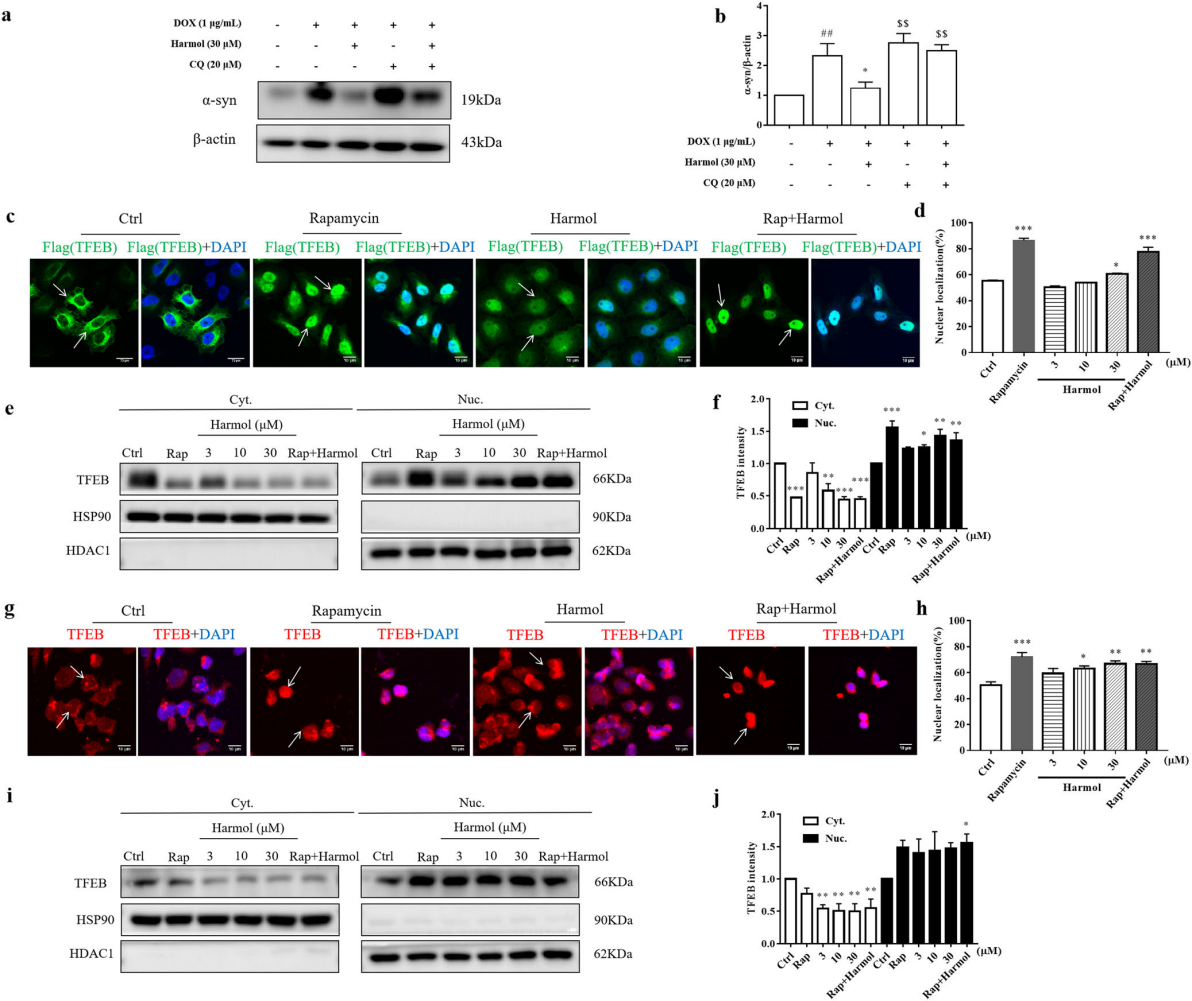
Harmol promotes the degradation of α-syn through the autophagy-lysosome pathway and the nuclear translocation of TFEB. **a** Cells were treated with harmol in the presence or absence of CQ (20 µM) for 24 h. The expression of α-syn was determined by western blot. **b** Data are presented as the mean ± SEM from three independent experiments. ^##^*P* < 0.01 vs. the control (0.1% DMSO). ^*^P < 0.05 vs. the DOX. ^$$^*P* < 0.01 vs. the DOX + harmol. **c** HeLa cells stably expressing 3xFlag-TFEB were treated with harmol (30 µM), rapamycin (Rap, 0.25 µM), and the combined administration of harmol (30 µM) and rapamycin (0.25 µM) for 24 h. Cells were fixed and stained with anti-Flag M2 (green) and DAPI (blue). **d** Data are presented as the mean ± SEM from three independent experiments. ^*^P < 0.05 and ^***^P < 0.001 vs. the control (0.1% DMSO). **e** The levels of 3xFlag-TFEB in the cytosolic (Cyt.) and nuclear (Nuc.) fractions were determined by western blot. **f** Data are presented as the mean ± SEM from three independent experiments. ^*^P < 0.05, ^**^P < 0.01 and ^***^P < 0.001 vs. the control (0.1% DMSO). **g** N2a cells were treated with harmol then fixed and stained with TFEB antibody (bred) and DAPI (blue). **h** Data are presented as the mean ± SEM from three independent experiments. ^*^P < 0.05, ^**^P < 0.01 and ^***^P < 0.001 vs. the control (0.1% DMSO). **i** The levels of endogenous TFEB in the cytosolic (Cyt.) and nuclear (Nuc.) fractions were determined by western blot. **j** Data are presented as the mean ± SEM from three independent experiments. ^*^P < 0.05 and ^**^P < 0.01 vs. the control (0.1% DMSO).

To identify whether harmol promotes α-syn clearance through ALP, cells were co-treated with harmol and autophagy-lysosome inhibitor chloroquine (CQ). As shown in Fig. 2a, b, CQ blocks harmol-induced degradation of α-syn. This result suggests that harmol might promote the clearance of α-syn via ALP.

TFEB, a major regulator of autophagy and lysosomal biogenesis, translocates into the nucleus where it promotes the transcription of autophagic and lysosomal genes^30^. To determine whether harmol induces nuclear translocation of TFEB, we used effective mTOR inhibitor rapamycin as a positive control. In normal conditions, the exogenous TFEB of HeLa cells stably expressing 3xFlag-TFEB locates in the cytoplasm (Fig. 2c, d). Harmol (30 μM) induces ~60% Flag-TFEB nuclear translocation (Fig. 2c, d). When harmol and rapamycin were co-administered simultaneously, we found that there was no significant additive effect on the nuclear translocation of TFEB (Fig. 2c, d). Quantification of TFEB levels in the cytosolic and nuclear fractions by Western blot further demonstrated that harmol significantly promotes nuclear translocation of exogenous TFEB (Fig. 2e, f). In addition, the effect of harmol on the translocation of endogenous TFEB in N2a cells was simultaneously determined by a high-content assay and western blot. Similarly, harmol significantly promotes the translocation of endogenous TFEB to the nucleus (Fig. 2g–j). These results indicate that harmol may be a novel TFEB activator.

### Harmol promotes autophagy flux and lysosomal biogenesis

**Fig. 3:**
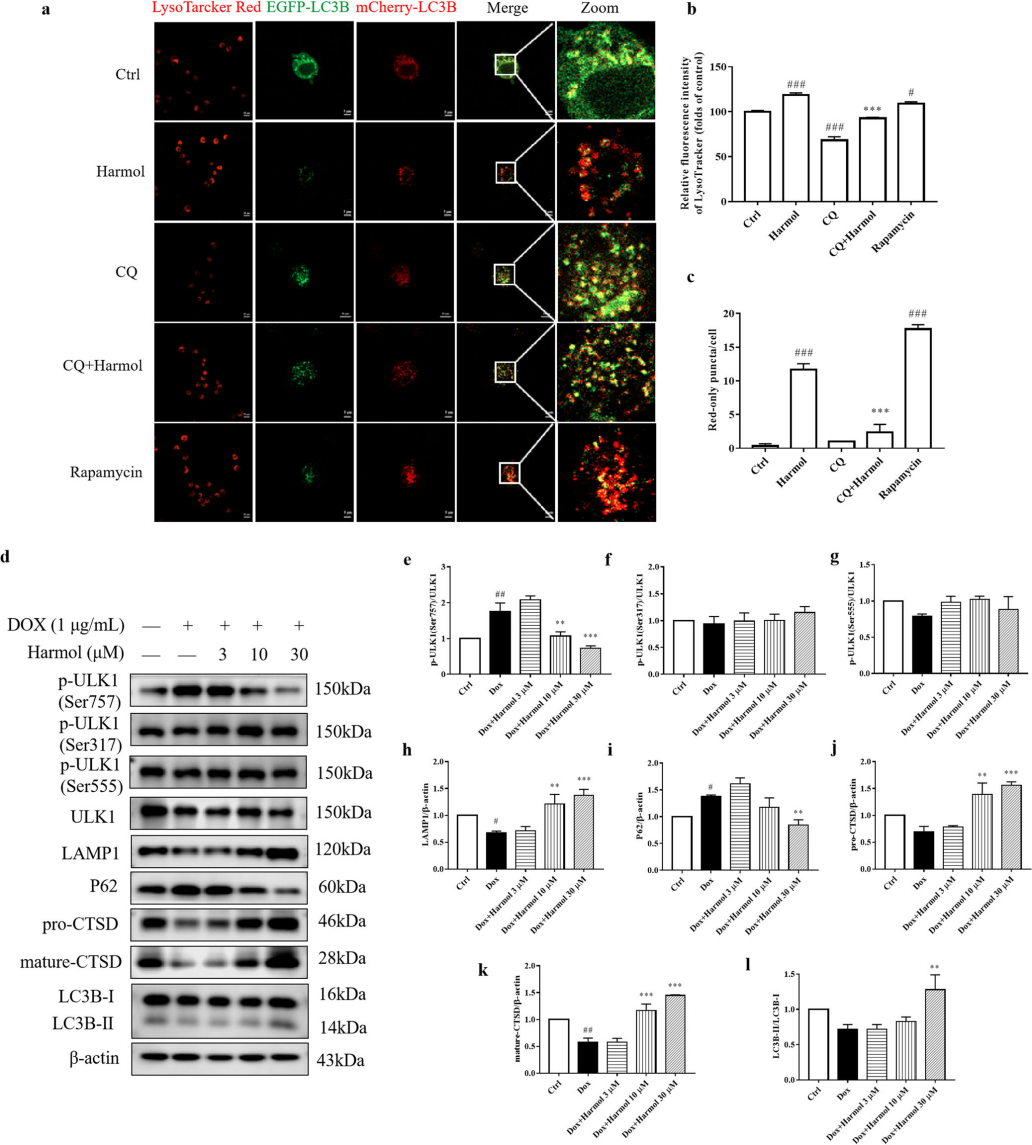
Harmol promotes autophagy flux and lysosomal biogenesis. **a** N2a cells were treated with the indicated compounds (harmol, 30 µM; CQ, 50 µM; rapamycin, a positive control, 0.25 µM) for 24 h, and then stained with Lyso-Tracker Red (50 nM) for 1 h. In addition, after treated for 2 h, N2a cells were transiently transfected with mCherry-GFP-LC3B adenovirus. Representative images are shown. **b** Fluorescence intensity was quantified as the mean ± SEM from three independent experiments. ^#^*P* < 0.05 and ^###^*P* < 0.001 vs. the control (0.1% DMSO). ^***^P < 0.001 vs. the harmol. **c** The numbers of red-only puncta per cell were quantified as the mean ± SEM from three independent experiments. ^###^*P* < 0.001 vs. the control (0.1% DMSO). ^***^P < 0.001 vs. the harmol. **d** PC12 inducible cells were treated with 1 µg/mL DOX for 24 h, then cells were incubated with 3, 10, and 30 µM harmol for 24 h. The levels of the LC3B-II/LC3B-I, p62, LAMP1, pro-CTSD, mature-CTSD, p-ULK1 (Ser757), p-ULK1 (Ser317), p-ULK1 (Ser555) and ULK1 were determined by western blot. Representative blots are shown. The expression of p-ULK1(Ser757)/ULK1 (**e**), p-ULK1(Ser317)/ULK1 (**f**), p-ULK1(Ser555)/ULK1 (**g**), LAMP1 (**h**), p62 (**i**), pro-CTSD (**j**), mature-CTSD (**k**), and LC3B-II/LC3B-I (**l**) were quantified as the mean ± SEM from three independent experiments. ^#^*P* < 0.05 and ^##^*P* < 0.01 vs. the control (0.1% DMSO). ^**^P < 0.01 and ^***^P < 0.001 vs. the DOX.

After translocation to the nucleus, TFEB triggers a transcriptional program activating multiple genes implicated in autophagy and lysosomal biogenesis. Since harmol increases the nuclear translocation of TFEB, we analyzed the effect of harmol on autophagy marker LC3B (microtubule-associated protein 1 light chain 3 β) to verify the effect of harmol on autophagy flux and lysosomal biogenesis. Harmol treatment significantly enhances LC3B-II/LC3B-I in PC12 inducible cells (Fig. 3d, l). Furthermore, degradation of p62, an autophagy substrate protein, indicates autophagic flux. Harmol also promotes autophagic degradation of p62 in PC12 inducible cells (Fig. 3d, i). To further confirm that harmol indeed enhances autophagy flux rather than lysosomal stress, we transfected N2a cells with the mCherry-EGFP-LC3B construct. Harmol and rapamycin significantly increase the number of red-only puncta, whereas CQ treatment increases the yellow puncta (Fig. 3a, c). CQ inhibits the effect of harmol (Fig. 3a, c). This suggests that harmol promotes autophagy flux and increased lysosomal degradation. In addition, we explored the effect of harmol on lysosomal biogenesis and found that harmol significantly increases the lysosome contents, as determined by Lyso-Tracker Red staining (Fig. 3a, b). Harmol dramatically increases the expression of lysosome marker LAMP1 (lysosomal-associated membrane protein 1), the precursor (pro-, 46 kDa), and mature (mature-, 28 kDa) forms of CTSD (cathepsin D) in PC12 inducible cells (Fig. 3d, h, j, k). Moreover, the phosphorylation of ULK1 (unc-51 like kinase 1) at Ser757 involved in autophagosome formation is decreased by harmol, and then initiated autophagy (Fig. 3d, e). However, no significant differences are detected in the phosphorylation of ULK1 at Ser317 and Ser555 (Fig. 3d, f, g).

### Harmol activates AMPK-mTOR-TFEB pathway

**Fig. 4:**
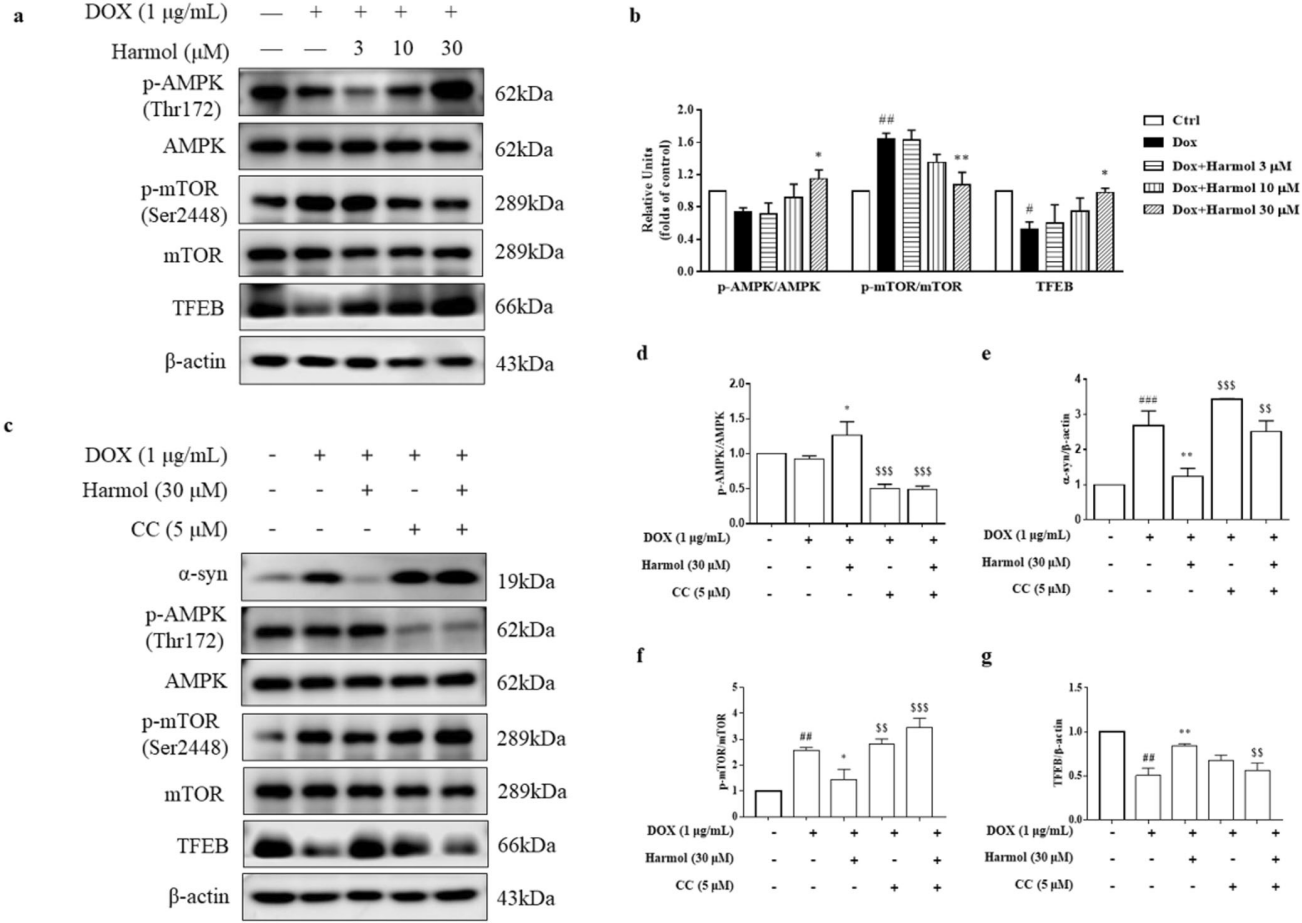
Harmol activates the AMPK-mTOR-TFEB pathway. **a** PC12 inducible cells were induced by 1 µg/mL DOX for 24 h, and cells were incubated with 3, 10, and 30 µM harmol for 24 h. Protein extracts were subjected to Western blot analysis. Representative blots are shown. **b** The levels of p-AMPK/AMPK, p-mTOR/mTOR and TFEB were quantified as the mean ± SEM from three independent experiments. ^#^*P* < 0.05 and ^##^*P* < 0.01 vs. the control (0.1% DMSO). ^*^P < 0.05 and ^**^P < 0.05 vs. the DOX. **c** Cells were treated with 30 µM harmol in the presence or absence of CC (5 µM), and representative blots are shown. The levels of p-AMPK/AMPK (**d**), α-syn (**e**), p-mTOR/mTOR (**f**), and TFEB (**g**) were quantified as mean ± SEM from three independent experiments. ^##^*P* < 0.01 and ^###^*P* < 0.001 vs. the control (0.1% DMSO). ^*^P < 0.05 and ^**^P < 0.01 vs. the DOX. ^$$^*P* < 0.01 and ^$$$^*P* < 0.001 vs. the DOX + harmol.

Since harmol activates TFEB and promotes autophagy flux and lysosomal biogenesis, we next determined which signaling pathway is involved in harmol-induced TFEB activation. A key upstream regulator of TFEB is mTOR, and mTOR is partially regulated by AMPK. Western blot analysis revealed that harmol significantly promotes TFEB expression (Fig. 4a, b) and AMPK phosphorylation at Thr172 in a dose-dependent manner (Fig. 4a, b), although the phosphorylation of mTOR at Ser2448 was lower compared with the DOX group (Fig. 4a, b). To understand the role of AMPK activation in harmol-induced α-syn degradation and TFEB activation, AMPK inhibitor compound C (CC) was co-treated with harmol. Indeed, harmol-induced AMPK phosphorylation is blocked by CC (Fig. 4c, d), indicating that AMPK was successfully inhibited. Interestingly, CC also blocks harmol-induced α-syn degradation, mTOR inhibition, and TFEB upregulation (Fig. 4c, e–g). These findings suggest that harmol promotes α-syn clearance via the AMPK-mTOR-TFEB pathway.

### Harmol rescues behavioral deficits in A53T α-syn mice

**Fig. 5:**
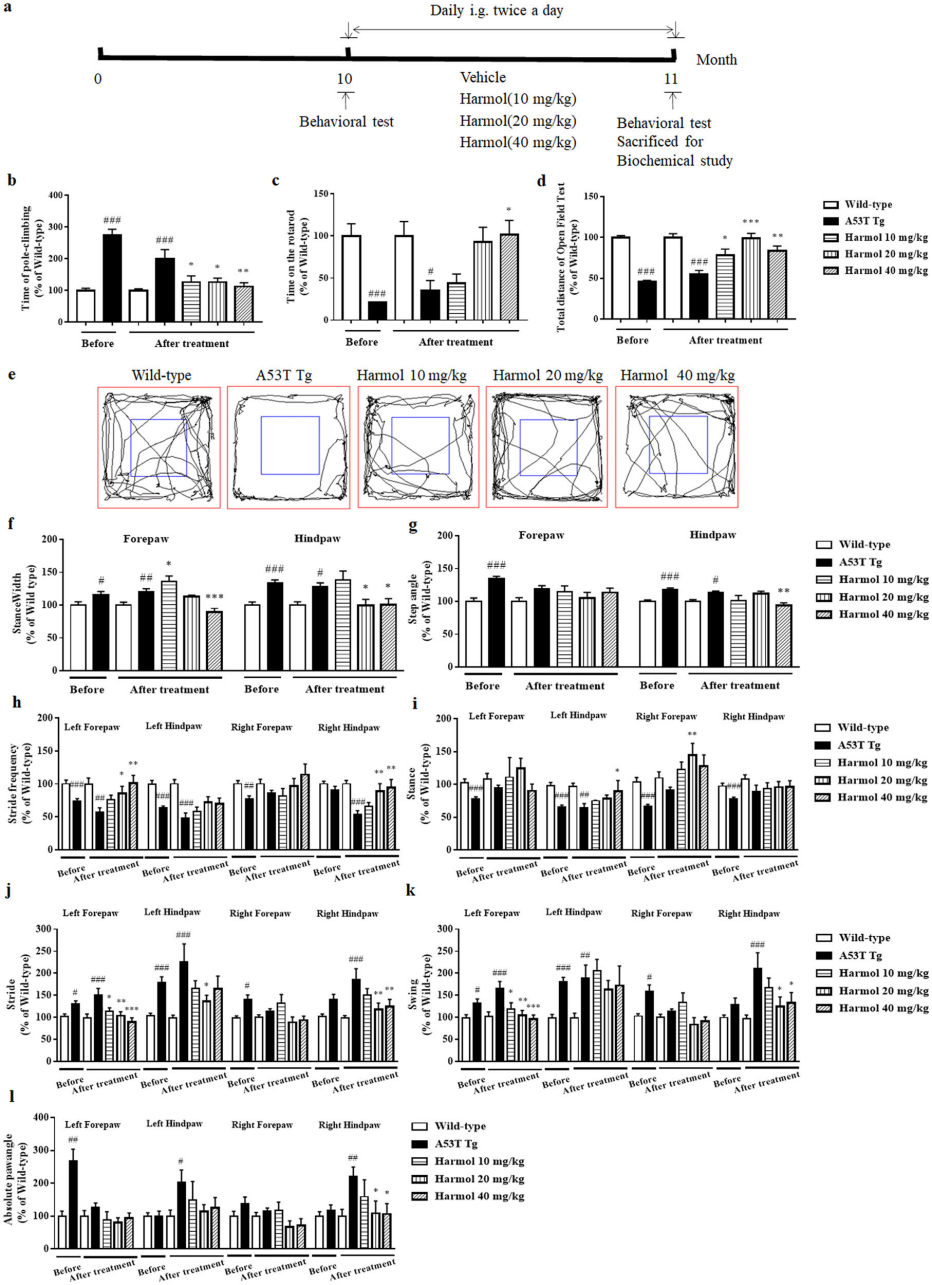
Harmol rescues behavioral deficits in A53T α-syn mice. **a** Experimental protocol for A53T α-syn mice. **b** Pole test. Quantification of the pole-climbing time for each treatment (mean ± SEM). ^###^*P* < 0.001 vs. the wild-type (saline). ^*^P < 0.05 and ^**^P < 0.01 vs. the A53T Tg. **c** Rotarod test. Quantification of the latency time on the rotarod for each treatment (mean ± SEM). ^#^*P* < 0.05 and ^###^*P* < 0.001 vs. the wild-type (saline). ^*^P < 0.05 vs. the A53T Tg. **d** Open-field test. Quantification of the total distance traveled in the open-field test for each treatment (mean ± SEM). ^###^*P* < 0.001 vs. the wild-type (saline). ^*^P < 0.05, ^**^P < 0.01, and ^***^P < 0.001 vs. the A53T Tg. **e** Representative exploratory patterns of mice in each group. **f**–**l** Automated treadmill gait test. Quantification of the stance width (**f**), step angle (**g**), stride frequency (**h**), stance (**i**), stride (**j**), swing (**k**), and absolute paw angle (**l**) for each treatment (mean ± SEM). ^#^*P* < 0.05, ^##^*P* < 0.01 and ^###^*P* < 0.001 vs. the wild-type (saline). ^*^P < 0.05, ^**^P < 0.01 and ^***^P < 0.001 vs. the A53T Tg.

Increasing evidence suggests α-syn inclusion causes neurodegeneration and produces severe and complex motor impairment leading to paralysis and death in mice expressing A53T mutant human α-syn^31^. In this study, we used multiple behavioral tests to evaluate motor performance, including the climbing-pole test, rotarod test, open-field test, and treadmill gait test. We first determined the onset of disease in A53T α-syn mice. At 10 months of age, A53T α-syn mice had longer pole-climbing time than wild-type mice of the same age (Fig. 5b). Furthermore, the latency time on the rotarod and the distance traveled during the open-field test were significantly reduced in A53T α-syn mice (Fig. 5c–e), suggesting A53T α-syn mice had decreased autonomous motor behavior. A53T α-syn mice also had an abnormal gait, including increased stance width, step angle, stride, swing, and absolute paw angle, as well as decreased stride frequency and stance (Fig. 5f–l).

To investigate the possibility that harmol rescues behavioral deficits of α-syn pathology, 10-month-old A53T α-syn transgenic mice received intragastric administration of harmol for 1 month (Fig. 5a). In the climbing-pole test, these mice exhibited faster pole-climbing times after 1 month of harmol treatment compared to A53T α-syn mice (Fig. 5b). Similarly, harmol-treated mice lasted longer on the accelerating rotarod (Fig. 5c), traveled greater distances in the open-field test, and demonstrated restored autonomous motor behavior (Fig. 5d, e) compared with A53T α-syn mice. We also used the automated DigiGait treadmill apparatus to gauge gait parameters of these mice. A53T α-syn mice initially demonstrated decreased stride frequency and stance, although both these parameters increased following harmol treatment (Fig. 5h, i). At the same time, harmol treatment significantly decreased the stance width, step angle, stride, swing, and absolute paw angle of compared to A53T α-syn mice (Fig. 5f, g, j–l). Accordingly, harmol ameliorates motor deficits of the mice including motor and coordination, autonomous motor behavior, and gait.

### Harmol attenuates α-syn and p62 load in the substantia nigra and prefrontal cortex of A53T α-syn mice

**Fig. 6:**
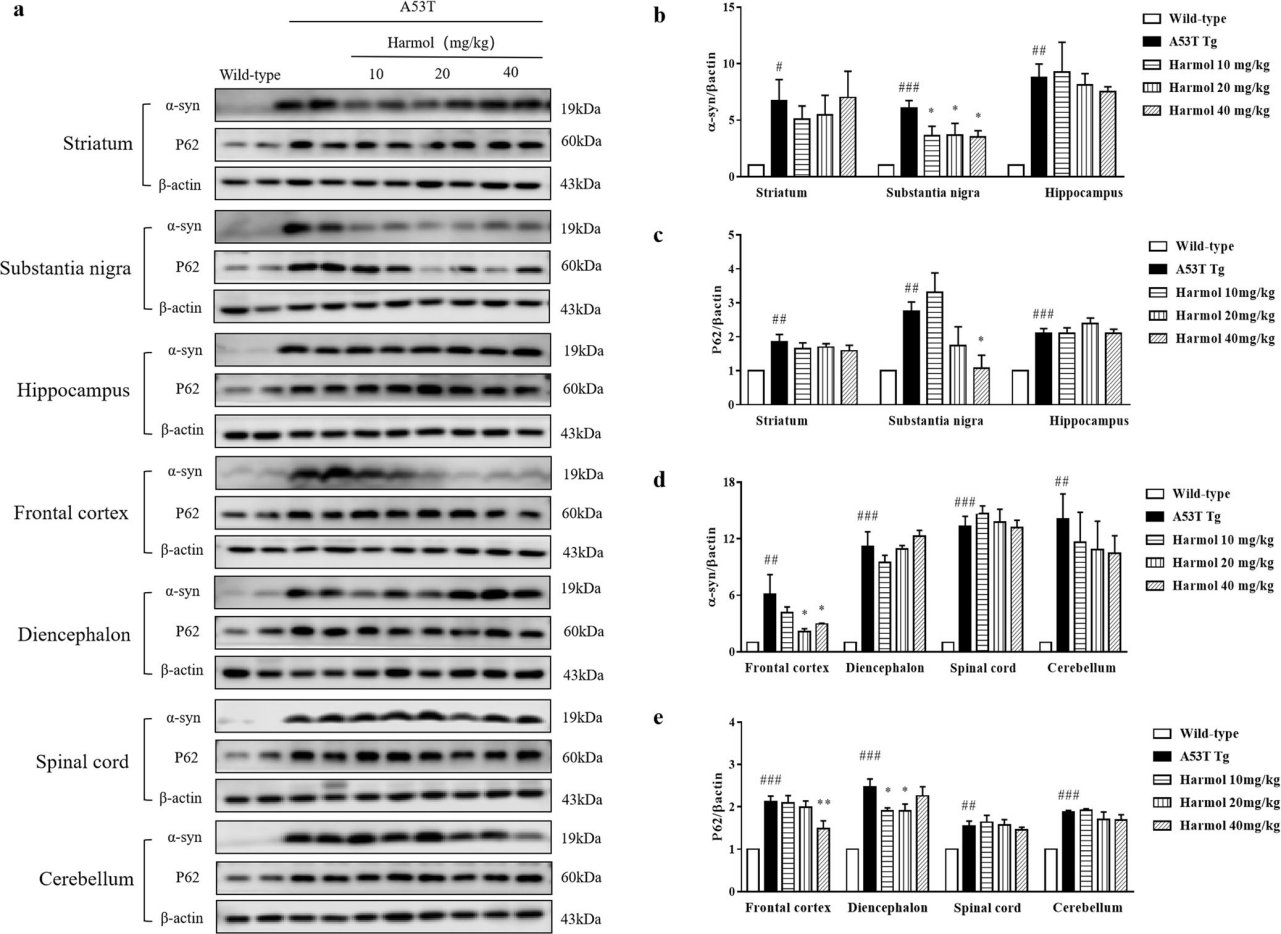
Harmol attenuates α-syn and p62 load in the substantia nigra and prefrontal cortex of A53T α-syn mice. **a** A53T α-syn mice were treated with 10, 20, and 40 mg/kg harmol for 1 month. The levels of α-syn and p62 in different brain regions were determined by western blot. Representative blots are shown. **b**–**e** Relative intensity was normalized to that of β-actin. Data are presented as the mean ± SEM. ^#^*P* < 0.05, ^##^*P* < 0.01 and ^###^*P* < 0.001 vs. the wild-type (saline). ^*^P < 0.05 vs. the A53T Tg.

A53T α-syn transgenic animals developed age-dependent intracytoplasmic neuronal α-syn inclusions paralleling disease onset, and the α-syn inclusions recapitulated features of human counterparts^31^. In addition, the inclusions were widely distributed throughout each brain region^31^. According to the Braak model, Lewy pathology started with the peripheral nervous system and gradually affected the central nervous system, which in turn included premotor, motor, and non-motor symptoms^32^. The functions of the substantia nigra, striatum^33^, spinal cord, and cerebellum^34^ might be related to the motor phenotypes, and hippocampus and prefrontal cortex might be related to the non-motor phenotypes^35^. The levels of α-syn and p62 expression in A53T α-syn mice were determined by western blot. As shown in Fig. 6a, b, d, harmol dose-dependently decreased the expression of α-syn in the substantia nigra and prefrontal cortex of the brain, while no differences were detected in other brain regions. This may be because α-syn is widely distributed, but the expression of α-syn is different in each brain region and the autophagic activities vary between these brain regions^36^. As shown in Fig. 6a, c, e, harmol dose-dependently decreased p62 in substantia nigra and prefrontal cortex, however in other brain regions, similar to α-syn, there were no significant difference. These observations suggest that harmol sufficiently promotes the degradation of α-syn inclusions in brain regions that play important roles in the complex motor and cognitive features of PD.

### Harmol activates the AMPK-mTOR-TFEB pathway and enhances autophagy and lysosome biogenesis in A53T α-syn mice

**Fig. 7:**
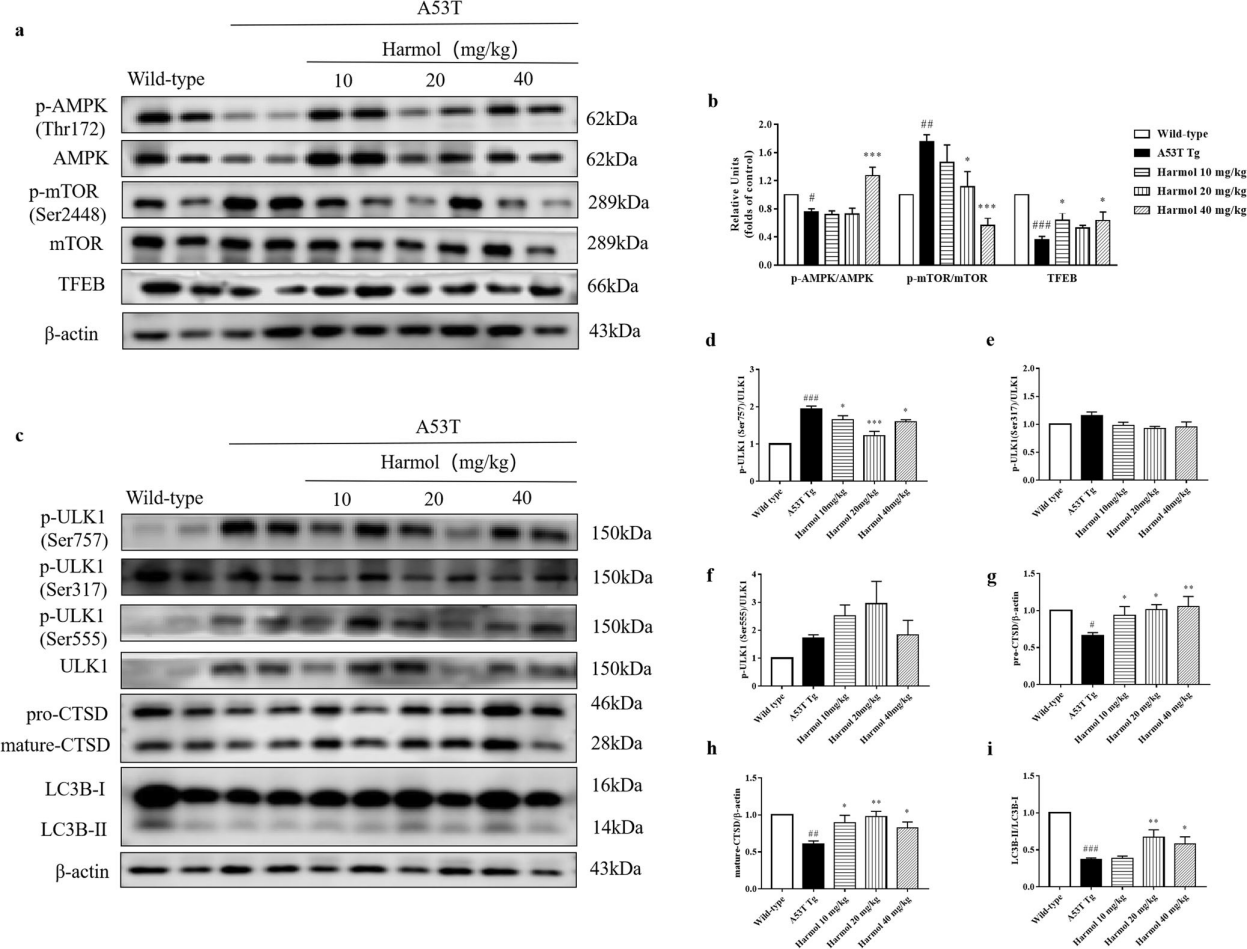
Harmol activates the AMPK-mTOR-TFEB pathway and enhances autophagy and lysosome biogenesis in A53T α-syn mice. **a** A53T α-syn mice were treated with 10, 20, and 40 mg/kg harmol for 1 month. Substantia nigra were homogenized and extracted for Western blot analysis. Representative blots of p-AMPK, AMPK, p-mTOR, mTOR, and TFEB are shown. **b** The levels of p-AMPK/AMPK, and p-mTOR/mTOR, and TFEB were quantified as the mean ± SEM. ^#^*P* < 0.05, ^##^*P* < 0.01 and ^###^*P* < 0.001 vs. the wild-type (saline). ^*^P < 0.05 and ^***^P < 0.001 vs. the A53T Tg. **c** A53T α-syn mice were administered with 10, 20, and 40 mg/kg harmol for 1 month. The levels of the LC3B-II/LC3B-I, pro-CTSD, mature-CTSD, p-ULK1 (Ser757), p-ULK1 (Ser317), p-ULK1 (Ser555) and ULK1 in substantia nigra were determined by western blot. Representative blots are shown. The expression of p-ULK1 (Ser757)/ULK1 (**d**), p-ULK1 (Ser317)/ULK1 (**e**), p-ULK1 (Ser555)/ULK1 (**f**), pro-CTSD (**g**), mature-CTSD (**h**), and LC3B-II/LC3B-I (**i**) was quantified as the mean ± SEM. ^#^*P* < 0.05, ^##^*P* < 0.01 and ^###^*P* < 0.001 vs. the wild-type (saline). ^*^P < 0.05, ^**^P < 0.01 and ^***^P < 0.001 vs. the A53T Tg.

In neuronal and non-neuronal cell cultures, harmol activates the AMPK-mTOR-TFEB pathway and enhances autophagy and lysosome biogenesis. Consistent with in vitro observations, the levels of AMPK phosphorylation at Thr172 and TFEB increased while the phosphorylation of mTOR at Ser2448 decreased in the substantia nigra after intragastric administration of harmol for 1 month (Fig. 7a, b). Meanwhile, harmol dose-dependently increased the expression of LC3B-II/LC3B-I, LAMP1, pro-CTSD, mature-CTSD and decreased the expression of p62 and p-ULK1(Ser757)/ULK1 in the substantia nigra (Fig. 7c, d, g–i). The levels of p-ULK1(Ser317)/ULK1 and p-ULK1(Ser555)/ULK1 in the substantia nigra were not obviously affected (Fig. 7c, e, f). These data confirm that harmol is sufficient to enhance autophagy-mediated degradation of protein aggregates in A53T α-syn mice.

## Discussion

Traditional dopamine replacement therapies diminish motor deficits in PD patients but lack neuroprotective and/or disease-modifying effects. An increased understanding of the etiopathogenesis of PD has led to the development of potential neuroprotective and/or disease-modifying therapies, including gene therapy^37^, immunotherapy, glucagon-like peptide 1 (GLP1) receptor agonists, iron chelators^38^, specific glucocerebrosidase (GBA) modifiers^39^ and leucine-rich repeat kinase 2 (LRRK2) inhibitors^40^. Immunotherapies including vaccination or monoclonal antibodies against aggregated and toxic α-syn are currently being investigated in clinical trials^41^. However, monoclonal antibodies do not always penetrate the blood-brain barrier and may be neutralized by the host. In addition, whether the antibodies reach their target at levels sufficient to produce the expected effect on the target protein and for how long and with what amplitude remains unknown^37^. Compared to these candidates, harmol, a smaller molecule, is more likely to reach the brain than monoclonal antibodies without the risk of being neutralized by host. Moreover, some autophagy inducers have widely shown the potential therapeutic effect for PD, such as rapamycin and metformin^42^. However, recent studies have provided convincing evidence that rapamycin treatment emerges the systemic tolerability side effects^43^. At the same time, metformin, the anti-diabetic drug, is a low-cost and generally well tolerated medication with minimal side effects. Even so, the prolonged consumption of metformin may induce the vitamin B12 deficiency and gastro-intestinal alterations, which can increase the risk of developing PD over time^44^. A natural product, harmol has fewer side effects and plays an important role in health maintenance and disease control. Finally, natural products can be used as lead compounds for structural modification to synthesize derivatives.

Traditional animal models of PD are designed using toxins, such as 6-hydroxydopamine (6-OHDA), rotenone, and 1-methyl-4-phenyl-1,2,3,6-tetrahydropyridine. Although toxin-treated models cause the dysfunction and death of dopaminergic neurons within substantia nigra, one of the major drawbacks of traditional models is the lack of Lewy body pathology^45^. A53T α-syn transgenic mice exhibit a full range of pathology including α-syn aggregation, oligomers, fibrils, phosphorylation, ubiquitination, and progressive neurodegeneration^45^. In this study, A53T α-syn transgenic animals developed the α-syn inclusions widely distributed throughout each brain region (Fig. 6), and the transgenic mice showed serious motor impairment (Fig. 5). In addition, the internalization and aggregation of misfolded α-syn can lead to ALP dysfunction in cellular and animal models or vice versa, suggesting that a crosstalk between α-syn aggregation and autophagic/lysosomal dysfunction may exist^46^. Our results prove that the overexpression of α-syn suppresses autophagic/lysosomal function (Figs. 3, 4, 6, and 7). However, the α-syn load did not affect the expression of dopaminergic neuron marker tyrosine hydroxylase (TH) within substantia nigra and striatum (Supplementary Fig. 2). Meanwhile, HPLC (high performance liquid chromatography) analysis showed no significant difference in total striatal DA, HVA (homovanillic acid), and DOPAC (3, 4-dihydroxyphenylacetic acid) content between wild-type and A53T α-syn transgenic mice (Supplementary Fig. 3). Increasing evidence suggests that mice overexpressing A53T α-syn do not lose dopaminergic neurons in substantia nigra, and motor deficits are caused by a loss of brain stem neurons and anterior horn motor neurons of the spinal cord^45^. In A53T α-syn transgenic mice, dopaminergic neurons do not show the same selective vulnerability that humans demonstrate. Why these neurons have different cross-species characteristics remains unclear, but the lack of neuromelanin formation in mice may be a determinant^31^. In addition, murine dopaminergic neurons display inexplicable resistance to α-syn-induced neurotoxicity compared with other neuronal populations^47^.

**Fig. 8:**
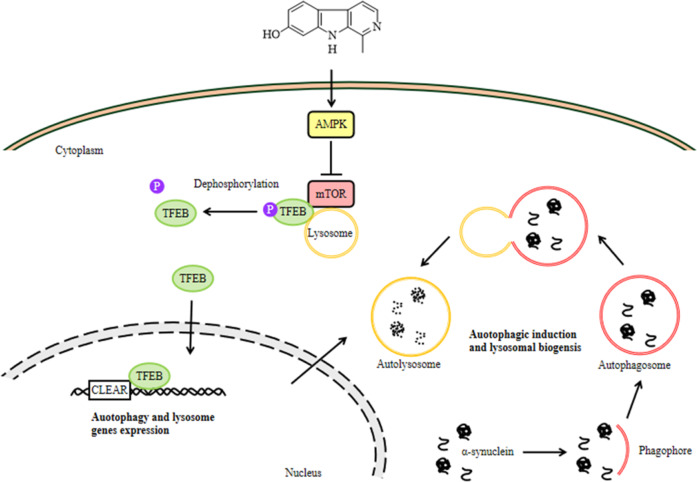
Underlying mechanisms by which harmol degrades α-syn. Harmol activates the AMPK and inhibits the mTOR. Cytoplasmic TFEB is highly phosphorylated and located in the lysosomal membrane, which binds with mTOR. Harmol inhibits the mTOR activity and promotes the dephosphorylation of TFEB. Dephosphorylated TFEB translocates into the nucleus, where it binds to the CLEAR sequence, leading to upregulation of the autophagic and lysosomal genes. Eventually, harmol enhances the autophagy-lysosome process and promotes the degradation of α-syn.

Autophagy and lysosome biogenesis could be enhanced by activating TFEB in vitro and in vivo^48^. Overexpressing TFEB or inducing its nuclear translocation stimulates ALP functioning and attenuates α-synuclein pathology^19^. In this study, we identified a natural ALP enhancer, harmol, that promotes the clearance of α-syn in vitro and in vivo (Figs. 1 and 6), and harmol-activated TFEB induces ALP (Figs. 2–4 and 7). AMPK promotes autophagy by inhibiting mTOR in the cytoplasm to allow nuclear translocation of TFEB^49^. Activation of AMPK-mTOR-TFEB axis-mediated autophagy promotes the clearance of toxic protein aggregates. Indeed, harmol administration increases phosphorylation of AMPK, inhibits phosphorylation of mTOR, and increases the expression of TFEB (Figs. 4 and 7). Furthermore, AMPK has been reported as an activator of autophagy via inhibition of the mTOR complex and direct phosphorylation and activation of ULK1. Specifically, AMPK can promote autophagy by directly activating ULK1 via phosphorylation of Ser317, Ser555, and Ser777^50^, while mTOR inhibits this process through ULK1 phosphorylation at Ser757. In this study, the p-ULK1(Ser757)/ULK1 is down-regulated, while p-ULK1(Ser317)/ULK1 and p-ULK1(Ser555)/ULK1 are not significantly affected (Figs. 3 and 7). This may indicate that AMPK activation mainly involves mTOR-ULK1 (Ser757) pathway in this study, which regulates autophagy. Compound C/Dorsomorphin is a primary reagent used as an AMPK inhibitor. Compound C can bind to phosphorylated-state mimic T172D mutant kinase domain of the human AMPKα subunit, and compound C can also block the AICAR cellular uptake by competing for adenosine transporter-binding sites^51^. Inhibition of the AMPK-mTOR-TFEB signaling pathway using AMPK blocker compound C diminishes the effects of harmol on autophagy and α-syn clearance (Fig. 4c–g). Hence, these studies suggest that harmol promotes α-syn clearance via the AMPK-mTOR-TFEB signaling pathway (Fig. 8).

Collectively, our research confirms that harmol activates the AMPK-mTOR-TFEB mediated ALP pathway in vitro and in vivo, contributing to the degradation of pathogenic proteins, the restoration of autophagic flux, and lysosomal biogenesis, and the improvement of motor impairment. These results increase the possibility that harmol may be developed into a therapeutic agent against aggregate-prone protein-associated neurodegenerative diseases such as PD, but there are still deficiencies and limitations. It is still not clear how harmol induces AMPK phosphorylation, and future work should aim to identify the upstream molecular signal or target for AMPK activation. Several studies have shown that TFEB also activates AMPK and, in turn, establishes a unique feed-forward loop between TFEB and AMPK^52^. Thus, harmol may promote autophagy via the APMK-mTOR-TFEB feed-forward loop, and future studies should test this possibility, although harmol may utilize other pathways to degrade α-syn or other mechanisms to regulate ALP.

## Methods

### Reagents and antibodies

Harmol was purchased from Tokyo Chemical Industry. Doxycycline was purchased from MedChemExpress. Dorsomorphin dihydrochloride was purchased from Targetmol. G418 sulfate was purchased from Amresco. Dopamine hydrochloride, homovanillic acid, 3, 4-dihydroxyphenylacetic acid, tri-sodium citrate dihydrate, citric acid, chloroquine, anti-Flag M2, and anti-α-synuclein antibodies were purchased from Sigma-Aldrich. Anti-TFEB antibody was purchased from Proteintech. Anti-tyrosine hydroxylase, anti-phospho-mTOR (Ser2448), anti-mTOR, anti-phospho-ULK1 (Ser757), anti-phospho-ULK1 (Ser317), anti-phospho-ULK1 (Ser555), anti-ULK1, anti-phospho-AMPKa (Thr172), anti-AMPKa, anti-LAMP1, anti-SQSTM1/p62, anti-TFEB, and anti-CTSD/cathepsin D antibodies were purchased from Cell Signaling Technology. Anti-phospho-α-synuclein and anti-LC3B antibodies were purchased from Abcam. 3-(4,5-dimethylthiazol-2-yl)-2,5-diphenyltetrazolium bromide was purchased from Beyotime Biotechnology.

### Animals and treatments

Male hA53T α-syn transgenic mice were obtained from Jackson Laboratory (Bar Harbor, ME, USA). Animals were propagated and fed at the Technology Industrial Park Development Co., Ltd of Guangzhou University of Chinese Medicine. The mice were housed under a 12 h light-dark cycle at 23 ± 2 °C and 60 ± 15% relative humidity. All animal care and experimental procedures were carried out in accordance with the guidelines of the Institutional Animal Care and Use Committee of Technology Industrial Park Development Co., Ltd of Guangzhou University of Chinese Medicine, and approved by the committee.

hA53T mice were randomly divided into four groups: saline (*n* = 6), harmol 10 mg/kg (*n* = 6), harmol 20 mg/kg (*n* = 6) and harmol 40 mg/kg (*n* = 6). Ten-month-old hA53T α-syn transgenic mice were treated with harmol (oral gavage twice daily) or an equal volume of saline for 1 month. Wild-type mice (*n* = 10) were administrated saline.

### Behavioral tests

To evaluate α-syn-induced behavioral deficits, the pole test, rotarod test, open-field test, and automated treadmill gait test was executed. All behavioral studies were double-blinded and repeated three times to obtain the average value for statistical analysis.

### Pole test

The climbing-pole test device was a metal rod that was ~50 cm high and 1 cm in diameter, wrapped with gauze to increase friction^53^. The mice were placed on top of the vertical pole facing down, and the descent time from top to bottom was recorded. Before the test, each mouse was trained for three consecutive days. The experiment was repeated three times in a row on the same day, and the average climbing time was calculated as the evaluation value.

### Rotarod test

For the rotarod test, the mice were trained for 3 consecutive days before the actual test^54^. Mice were placed on an accelerating rotarod cylinder, and the latency time was tested. The speed was slowly accelerated from 4 to 40 rpm within 4 min. The data are presented as the average latency time (three independent trials) on the rotarod.

### Open-field test

Animals were placed individually into the center of an acrylic apparatus (50 cm × 50 cm × 40 cm) and allowed to freely explore for 5 min under dim light. The apparatus was cleaned with 70% ethanol solution between each trial to avoid olfactory cues between animals. A video tracking system was used to record the total distance traveled as a measure of autonomous movement^55^.

### Automated treadmill gait test

Treadmill gait assessment was performed with the DigiGait imaging apparatus (Mouse Specifics Inc.)^56^. Mice were placed on a motorized treadmill within a plexiglass compartment (~25 cm long and 5 cm wide). A camera mounted under the transparent treadmill belt acquired digital video images at 80 frames per second to visualize paw contacts. The treadmill was set at a fixed speed of 15 cm/sec so that most animals could move continuously. Digital videos were analyzed by DigiGait software, which automatically recognized the pawprints, and if necessary, manual changes in the images were made to properly distinguish the pawprints from the background. Then, the images were automatically processed by the software to calculate the values of various gait parameters, including stride frequency, stride width, step angle, absolute paw angle, swing, stride, and stance.

### HPLC-ECD analysis

Striatum was homogenized on ice with 0.1 M perchloric acid. After centrifugation at 12,000 × *g* for 15 min, the supernatant was analyzed using an Agilent HPLC-ECD system (Agilent Technologies Inc., USA) with Agilent Eclipse plus C18 column (4.6 × 100 mm, 3.5 μm). The mobile phase was methanol (10%) and aqueous solution (90%, containing 13.764 g/L citric acid, 10.147 g/L tri-sodium citrate dihydrate, 269.25 mg/L sodium octane sulfonate, 37.2 mg/L ethylenediaminetetraacetic acid disodium salt dehydrate and 149.2 mg/L potassium chloride), and detection was carried out at 35 °C with a flow rate of 0.8 mL/min. For ECD, the detecting voltage was maintained at 750 mV, and the response range of the detector was set at 50 nA. The retention time of 3, 4-dihydroxyphenylacetic acid (DOPAC), dopamine (DA) and homovanillic acid (HVA) was 4.5, 6.2 and 10.3 min, respectively.

### Cell culture and drug treatment

N2a cells were provided by Prof. Simon MingYuen Lee (State Key Laboratory of Quality Research in Chinese Medicine and Institute of Chinese Medical Sciences, University of Macau, China) as a gift, and the cells were maintained in DMEM supplemented with 10% FBS. HeLa cells stably expressing 3xFlag-TFEB were supplied by Prof. Ju-Xian Song (Guangzhou University of Chinese Medicine, Guangzhou, China) as a gift, and cultured in DMEM containing 10% FBS and 500 μg/mL G418. Inducible PC12 cells were generously gifted from professor Yadong Huang (Jinan University, Guangzhou, China) and maintained in DMEM supplemented with 5% HS, 10% FBS, and 200 µg/mL G418. For drug treatment, the whole medium was replaced with a fresh medium containing the compounds and then incubated for the indicated periods.

### Cell viability

Cells were plated in a 96-well plate for 24 h. After 24 h of compound treatment, cell cytotoxicity was determined by 3-(4,5-dimethylthiazol-2-yl)-2,5-diphenyltetrazolium bromide (MTT) analysis, following the manufacturer’s guidelines. Absorbance was measured spectrophotometrically at 570 nm with a microplate reader (Tecan, Switzerland).

### High-content screening assay

To quantify TFEB subcellular localization, a high-content assay was performed using HeLa cells stably expressing 3xFlag-TFEB and N2a cells. Cells were seeded in 96-well plates for 24 h and treated with different concentrations of compounds. After 24 h, cells were fixed, permeabilized, blocked, and stained with anti-Flag M2 or anti-TFEB overnight at 4 °C. DAPI, Alexa Fluor^®^488 (green), or Alexa Fluor^®^ 594 (red) secondary antibodies were added for 2 h at room temperature. Images were acquired by confocal automated microscopy (IN Cell Analyzer 6000, USA). The ratio value of the average nuclear fluorescence intensity divided by the average cytosolic fluorescence intensity was calculated^57^. Data are represented by the percentage of nuclear translocation at different concentrations of each compound using GraphPad Prism 7 software (GraphPad Software, USA).

### Autophagy flux assay

N2a cells were transfected with mCherry-GFP-LC3B adenovirus for 24 h, and then the medium was replaced with a regular culture medium. After 48 h, the cells were respectively treated with harmol, CQ and rapamycin for 2 h. Images were obtained using a fluorescent confocal microscope (Zeiss, Germany) and were quantified by ImageJ software (Version 1.52, USA).

### Lyso-Tracker Red staining

Lysosome activity was evaluated using Lyso-Tracker Red (Beyotime Biotechnology, C1046) according to the manufacturer’s protocol. After 24 h of drug treatment, Lyso-Tracker Red (50 nM) was added for 1 h. The cells were washed three times with PBS, and observed using a fluorescent confocal microscope (Zeiss, Germany) and were quantified by ImageJ software (Version 1.52, USA).

### Western blotting analysis

Cells or animal tissues were lysed on ice with RIPA lysis buffer containing a protease phenylmethylsulfonyl fluoride and phosphatase inhibitor cocktail. Cytosolic and nuclear fractions were isolated using the Nuclear and Cytoplasmic Extraction Reagents (Thermo Scientific, 78833) according to the manufacturer’s protocol.

Proteins were separated by 8–12% sodium dodecyl sulfate-polyacrylamide gel electrophoresis and transferred onto polyvinylidene fluoride membranes. After blocking with 5% nonfat milk, the membrane was probed with the primary and secondary antibodies. The protein signals were visualized using an ECL kit and detected by an image reader (GE AI600, USA). All blots were processed in parallel and derive from the same experiment.

### Statistics

All experimental data are presented as mean ± standard error of the mean (SEM). Differences among groups (≥3 groups) were analyzed using one-way ANOVA or differences between two groups using Student’s *t* test by GraphPad Prism 7 software (GraphPad Software, USA). Values of *P* < 0.05 were considered statistically significant.

## Supplementary information

Supplementary information

## Data Availability

The data that support the findings of this study are available from the corresponding author upon reasonable request.
