# Supplementary material for: Harmol promotes α-synuclein degradation and improves motor impairment in Parkinson’s models via regulating autophagy-lysosome pathway
Source: NPJ Parkinsons Dis. 2022 Aug 6;8:100. doi: 10.1038/s41531-022-00361-4 (PMC9357076; doi:10.1038/s41531-022-00361-4)

Harmol promotes α-synuclein degradation and improves motor impairment in Parkinson's models via regulating autophagy-lysosome pathway

Jie Xu[1,†], Yun-Lin Ao[1,†], Chunhui Huang[2], Xiubao Song[3], Guiliang Zhang[2], Wei Cui[4], Yuqiang Wang [2], Xiao-Qi Zhang [1,*], Zaijun Zhang [2,*]

[1] Guangdong Provincial Engineering Research Center for Modernization of TCM, Guangdong Provincial Key Laboratory of Pharmacodynamic Constituents of TCM and New Drug Research, College of Pharmacy, Jinan University, Guangzhou 510632, P. R. China.

[2] Institute of New Drug Research, College of Pharmacy, Jinan University, Guangzhou 510632, P. R. China.

[3] Department of Rehabilitation, the First Affiliated Hospital, Jinan University, Guangzhou 510630, China.

[4] Ningbo Key Laboratory of Behavioral Neuroscience, Zhejiang Provincial Key Laboratory of Pathophysiology, School of Medicine, Ningbo University, Ningbo 315211, China.

[†] These authors contributed equally to this work.

*Correspondence: zaijunzhang@163.com, ORCID: 0000-0002-0690-1673; xqzhang74@hotmail.com, ORCID: 0000-0002-4436-0273

Results

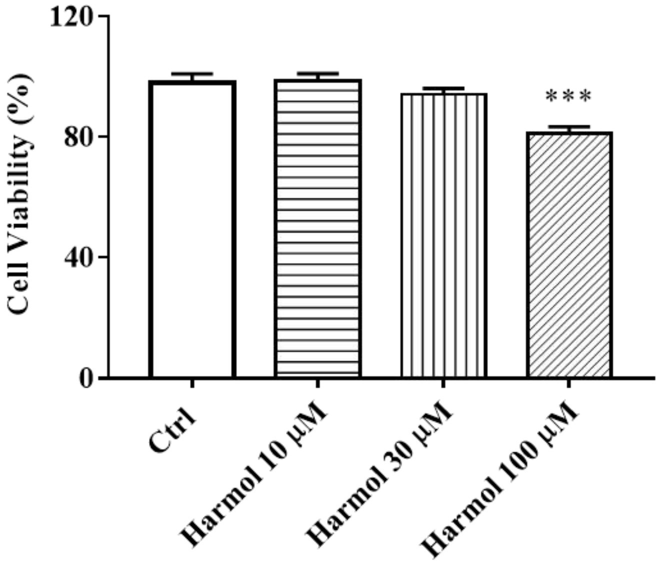

Supplementary Figure 1 The cell cytotoxicity of harmol

Tet-on inducible PC12 cells were plated in a 96-well plate for 24 h. After 24 h of harmol treatment, MTT reagent was added for another 4 h. Then, the absorbance was measured spectrophotometrically at 570 nm with a microplate reader. Data were quantified as the mean ± SEM from 3 independent experiments. $^{***}P<0.001$ vs. the control (0.1% DMSO).

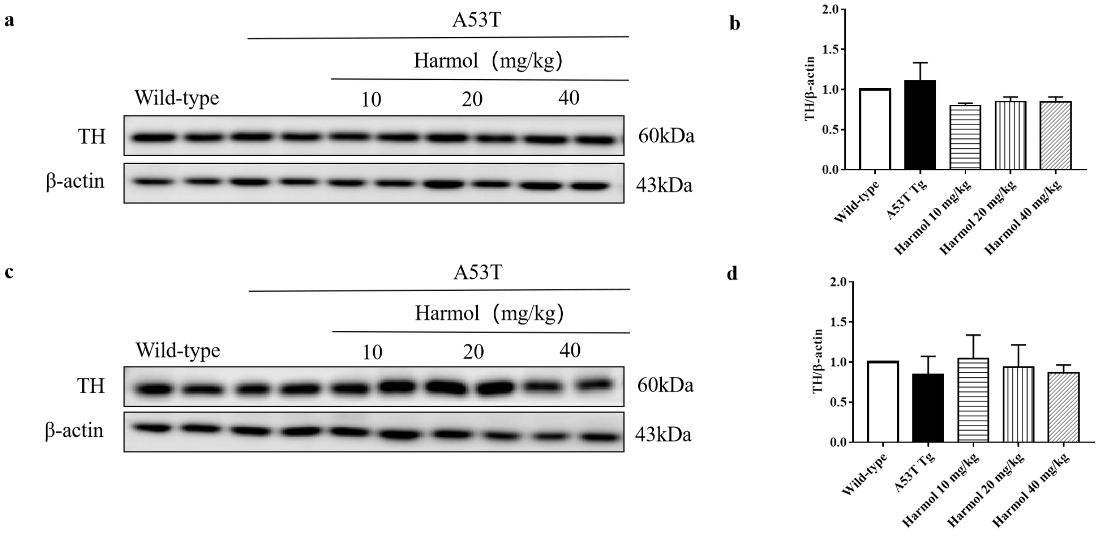

Supplementary Figure 2 The effect of harmol on dopaminergic neurons in substantia nigra and striatum of A53T α-syn mice

(**a**) A53T α-syn mice were treated with 10, 20, and 40 mg/kg harmol for 1 month. Striatum were homogenized and extracted for western blot analysis. Representative blots of TH are shown. (**b**) The levels of TH were quantified as mean ± SEM. (**c**) A53T α-syn mice were administered with 10, 20, and 40 mg/kg harmol for 1 month. Substantia nigra were homogenized and extracted for western blot analysis. Representative blots of TH are shown. (**d**) The levels of TH were quantified as mean ± SEM.

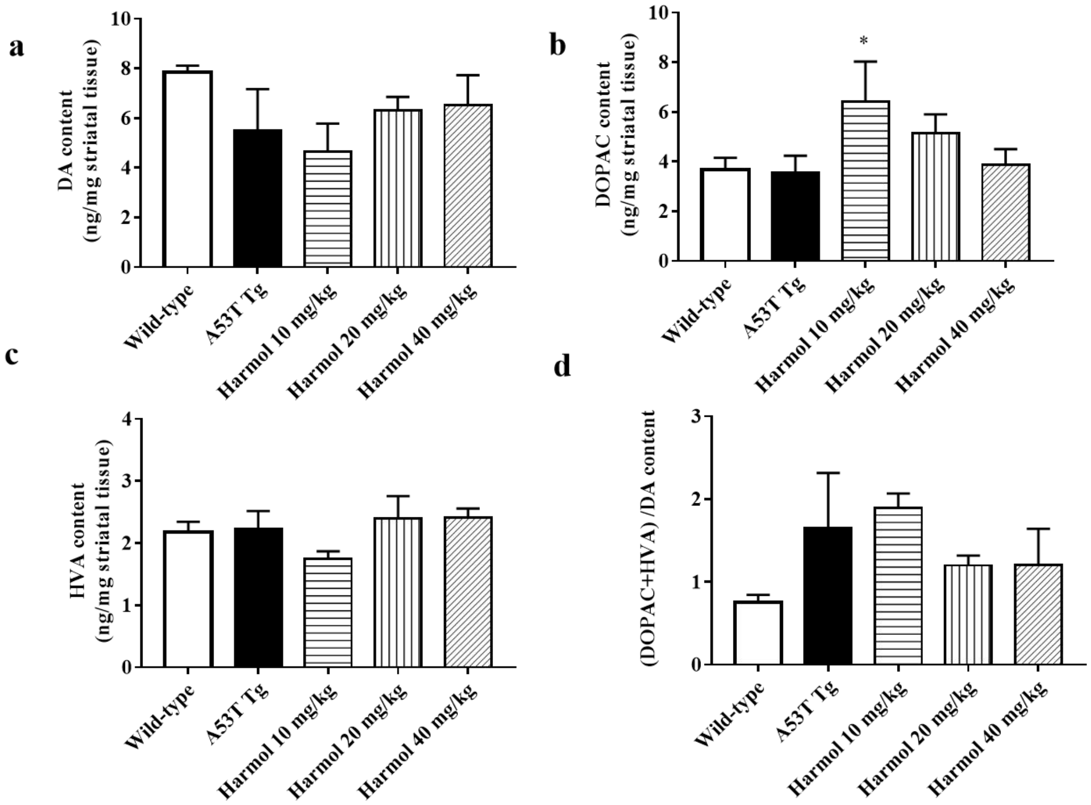

Supplementary Figure 3 The effect of harmol on DA, DOPAC and HVA in striatum of A53T α-syn mice

A53T α-syn mice were treated with 10, 20, and 40 mg/kg harmol for 1 month. Striatum samples were homogenized and extracted. Dopamine (**a**) and its main metabolites DOPAC (**b**), HVA (**c**) and (DOPAC+HVA)/DA (**d**) were determined by HPLC. Data were quantified as the mean ± SEM. *P<0.05 vs. the A53T Tg.

Western blots images

Fig.1b

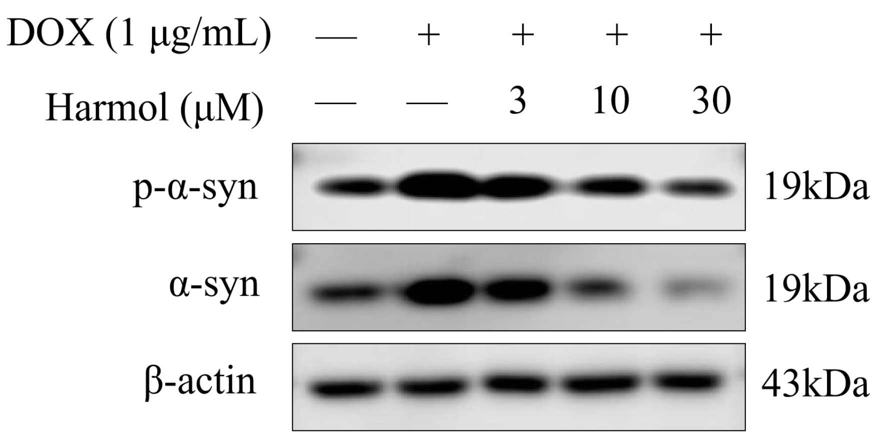

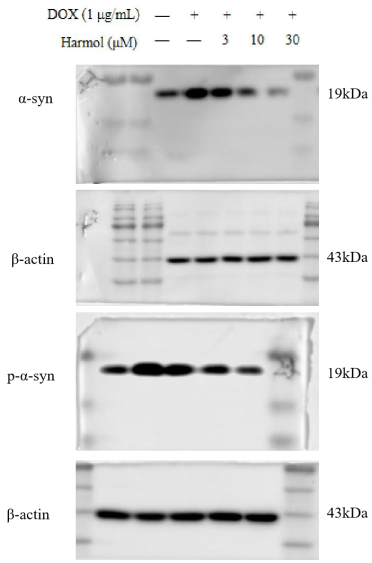

Fig.1d

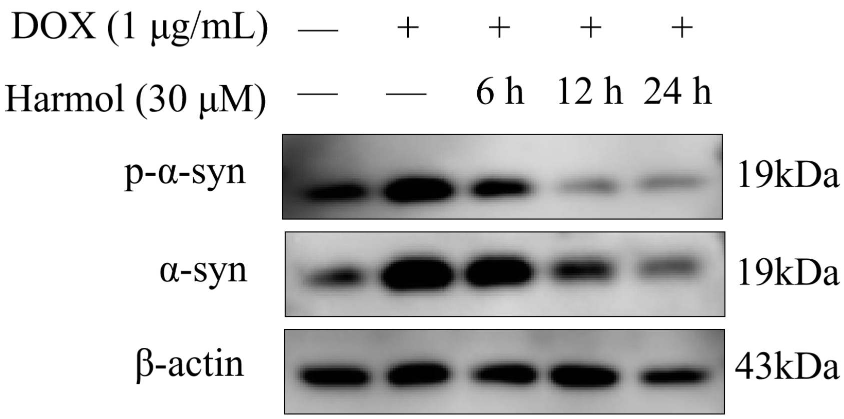
DOX (1 μg/mL) — + + + +
Harmol (30 μM) — — 6 h 12 h 24 h
p-α-syn
19kDa
α-syn
19kDa
β-actin
43kDa

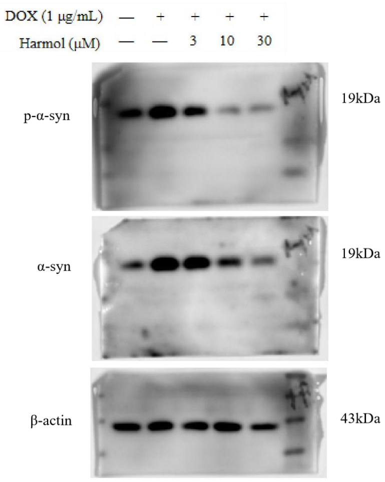
DOX (1 μg/mL) — + + + +
Harmol (μM) — — 3 10 30
p-α-syn
19kDa
α-syn
19kDa
β-actin
43kDa

Fig.2a

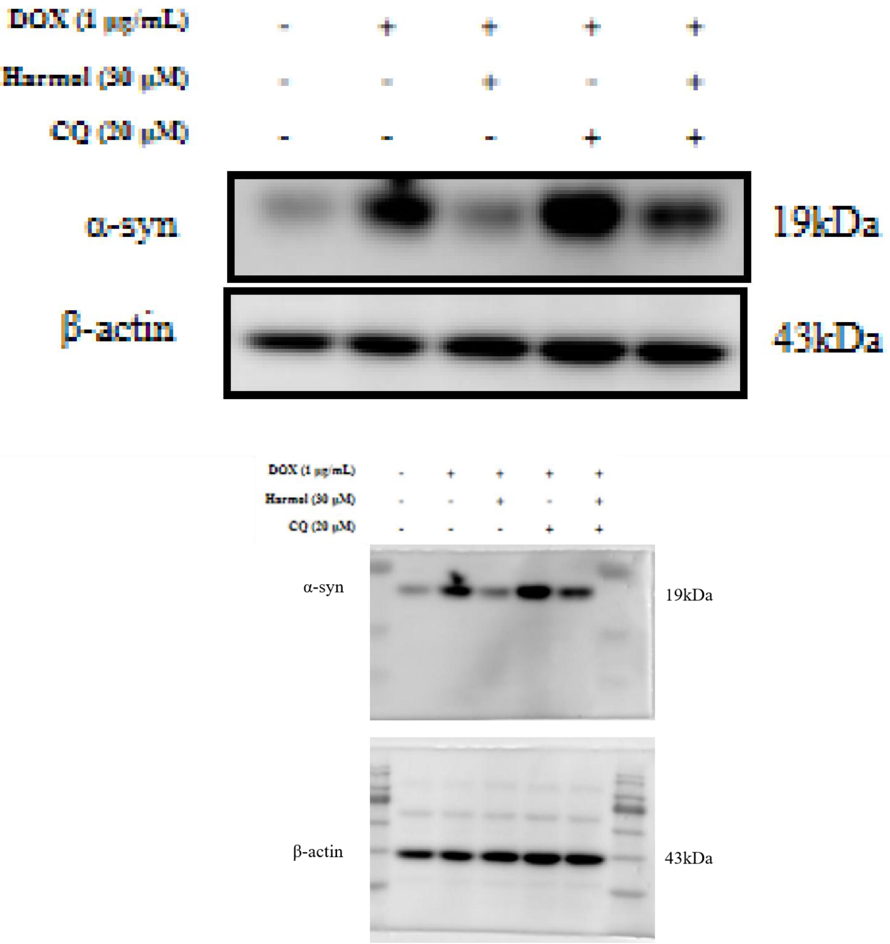

Fig.2e

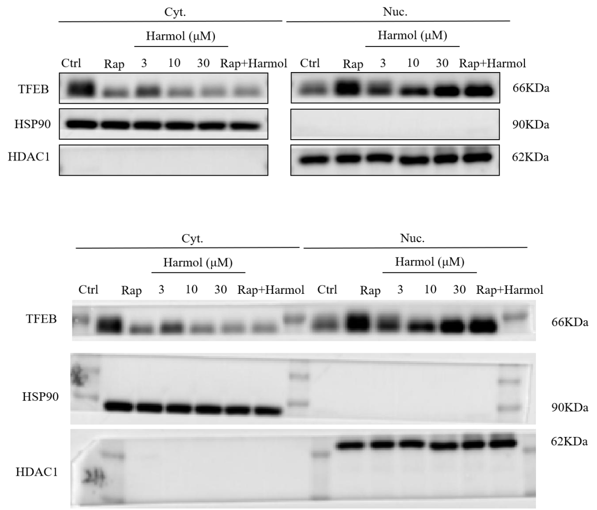

Fig.2i

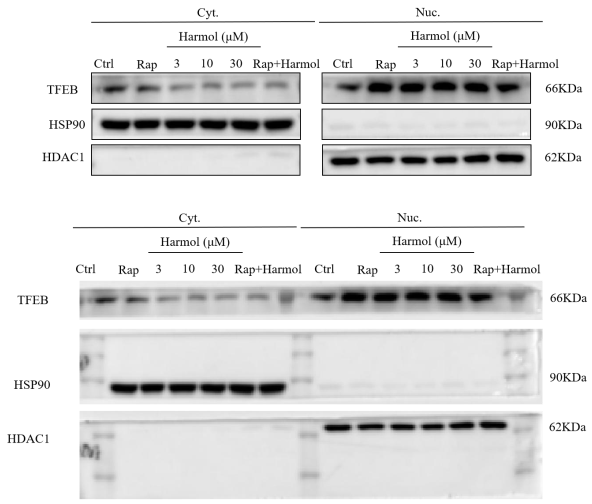

Fig.3d

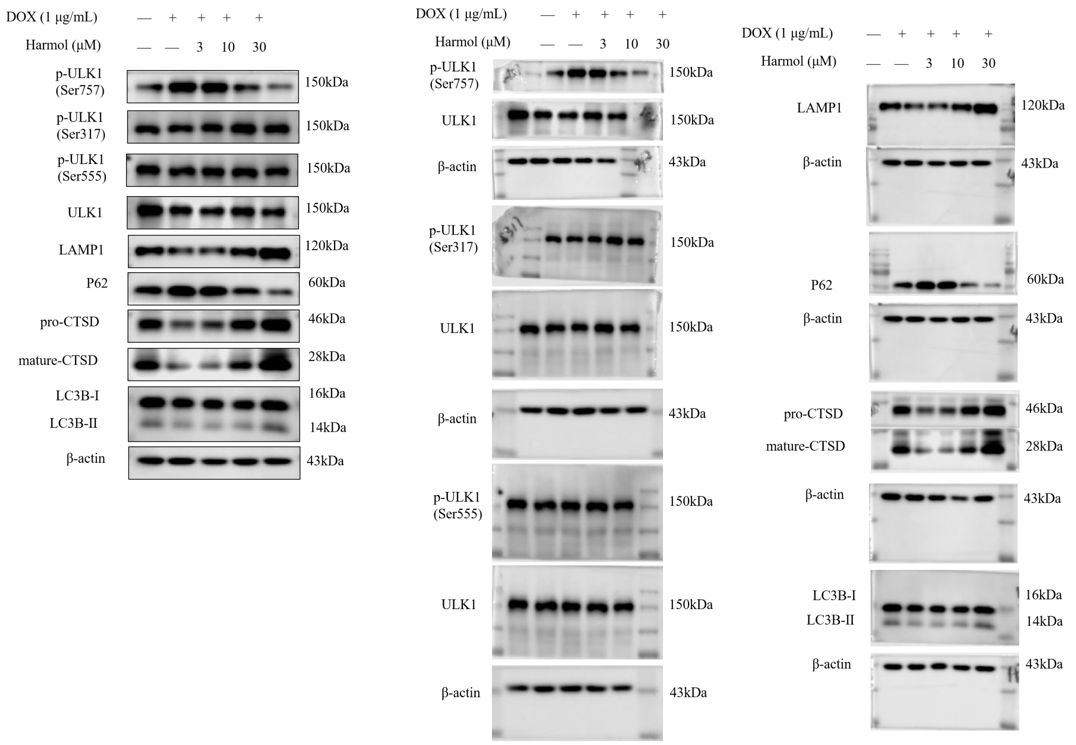
DOX (1 μg/mL) — + + + +
Harmol (μM) — — 3 10 30
p-ULK1 (Ser757)
p-ULK1 (Ser317)
p-ULK1 (Ser555)
ULK1
LAMP1
P62
pro-CTSD
mature-CTSD
LC3B-I
LC3B-II
β-actin
150kDa
120kDa
60kDa
46kDa
28kDa
16kDa
14kDa
43kDa

Fig.4a

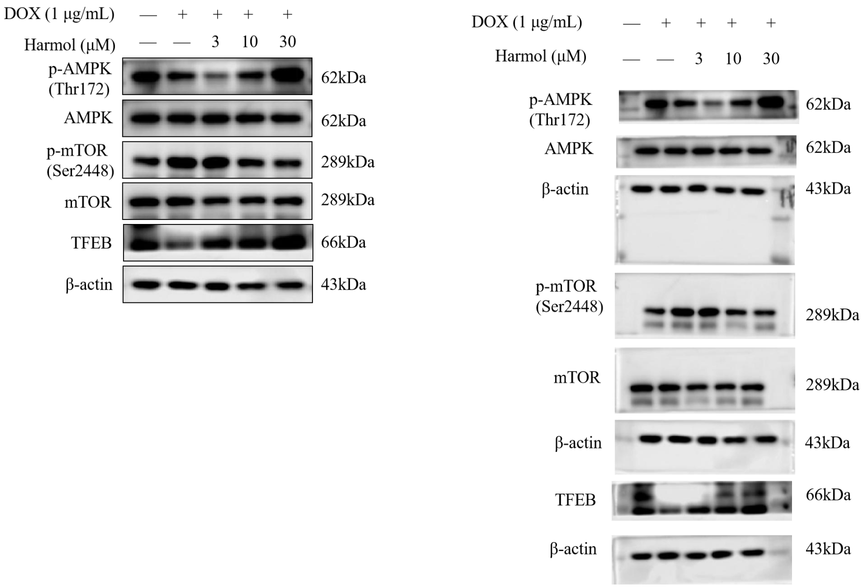
DOX (1 μg/mL) — + + + +
Harmol (μM) — — 3 10 30
p-AMPK (Thr172)
AMPK
p-mTOR (Ser2448)
mTOR
TFEB
β-actin
62kDa
289kDa
66kDa
43kDa

Fig.4c

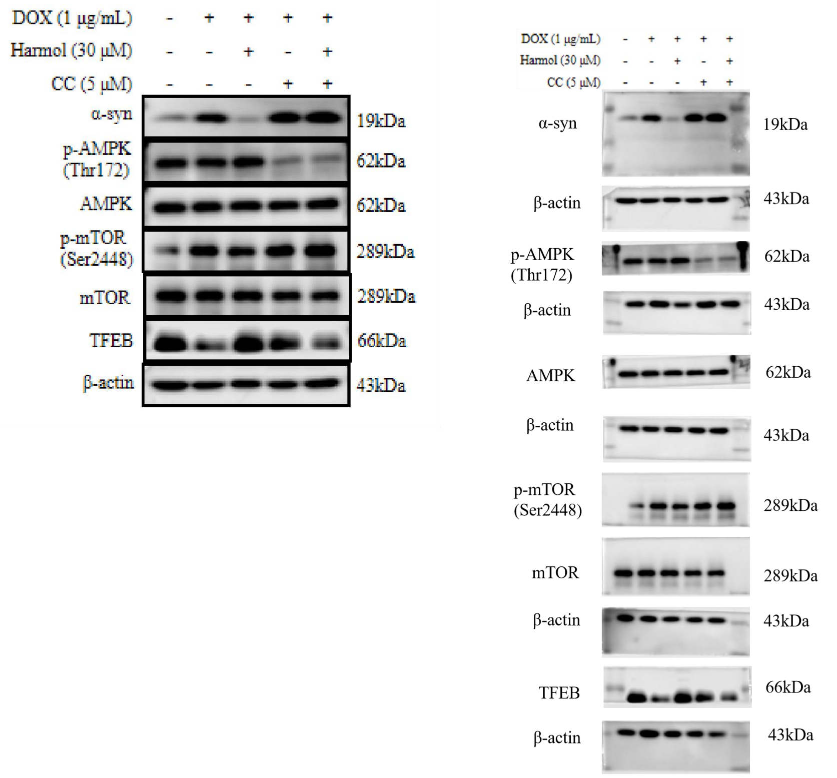
DOX (1 μg/mL)
Harmol (30 μM)
CC (5 μM)
α-syn
p-AMPK (Thr172)
AMPK
p-mTOR (Ser2448)
mTOR
TFEB
β-actin
19kDa
62kDa
62kDa
289kDa
289kDa
66kDa
43kDa
β-actin
43kDa

Fig.6a

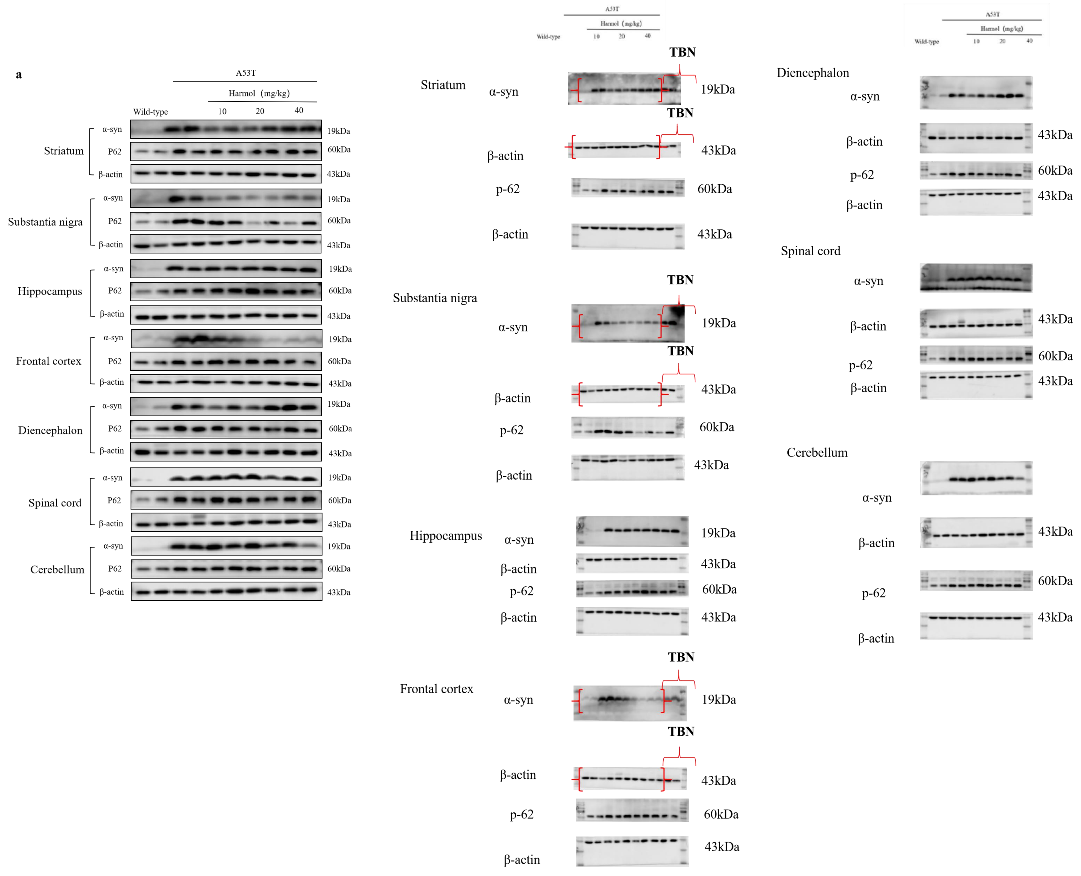
a
A53T
Harmol (mg/kg)
Wild-type
10
20
40
Striatum
Substantia nigra
Hippocampus
Frontal cortex
Diencephalon
Spinal cord
Cerebellum
α-syn
P62
p-62
β-actin
TBN
19kDa
60kDa
43kDa

Fig.7a

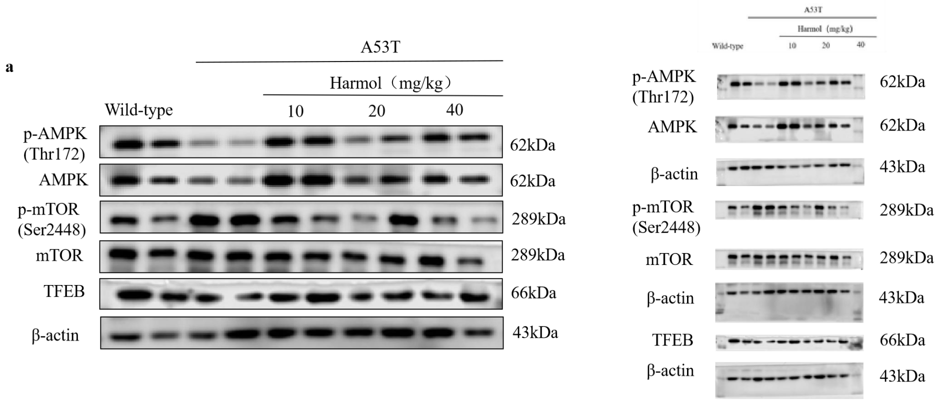
a
A53T
Harmol（mg/kg）
Wild-type
10
20
40
p-AMPK (Thr172)
AMPK
p-mTOR (Ser2448)
mTOR
TFEB
β-actin
62kDa
62kDa
289kDa
289kDa
66kDa
43kDa
p-AMPK (Thr172)
AMPK
β-actin
p-mTOR (Ser2448)
mTOR
β-actin
TFEB
β-actin
62kDa
62kDa
43kDa
289kDa
289kDa
43kDa
66kDa
43kDa

Fig.7c

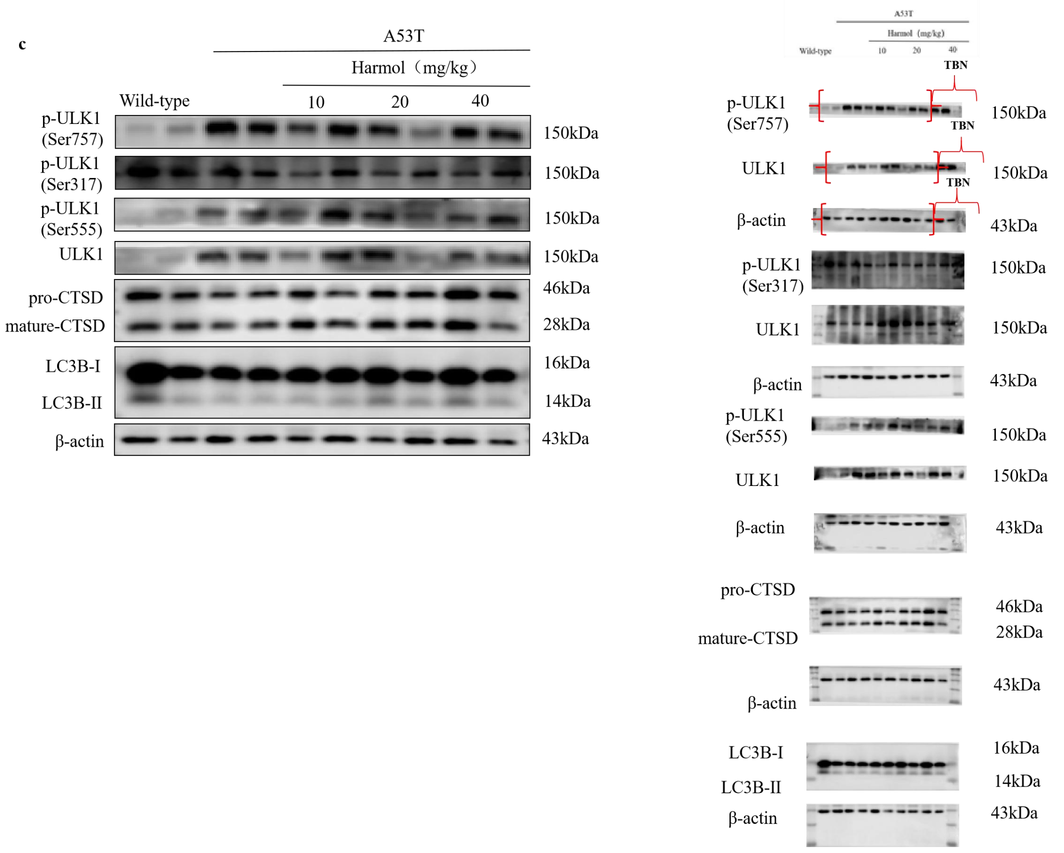
c
A53T
Harmol（mg/kg）
Wild-type
10
20
40
p-ULK1 (Ser757)
p-ULK1 (Ser317)
p-ULK1 (Ser555)
ULK1
pro-CTSD
mature-CTSD
LC3B-I
LC3B-II
β-actin
150kDa
150kDa
150kDa
150kDa
46kDa
28kDa
16kDa
14kDa
43kDa
TBN
p-ULK1 (Ser757)
ULK1
β-actin
p-ULK1 (Ser317)
ULK1
β-actin
p-ULK1 (Ser555)
ULK1
β-actin
pro-CTSD
mature-CTSD
β-actin
LC3B-I
LC3B-II
β-actin
150kDa
150kDa
43kDa
150kDa
150kDa
43kDa
150kDa
150kDa
43kDa
46kDa
28kDa
43kDa
16kDa
14kDa
43kDa

Supplementary Figure 2

a

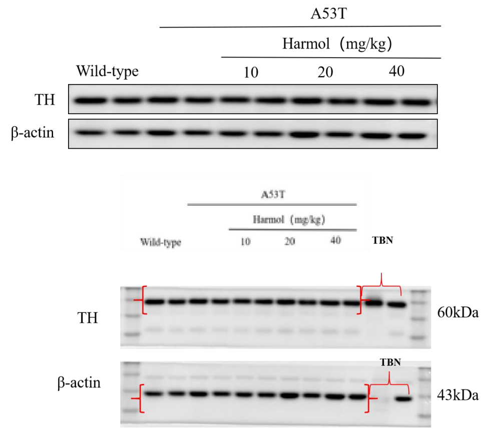

c

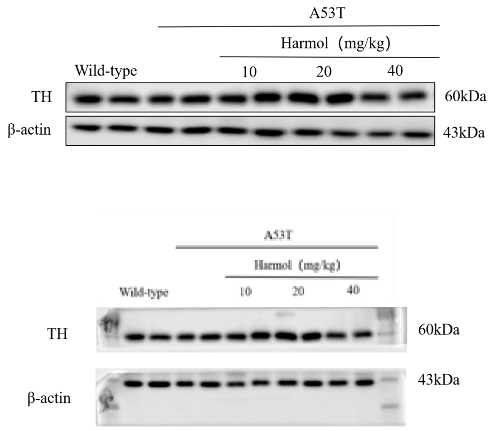

Supplement: Supplementary file 1 — Supplementary information [file 41531_2022_361_MOESM1_ESM.pdf]
